# Honeybee products and edible insect powders improve locomotive and learning abilities of *Ubiquilin-*knockdown *Drosophila*

**DOI:** 10.1186/s12906-020-03054-8

**Published:** 2020-08-31

**Authors:** Patcharin Phokasem, Salinee Jantrapirom, Jirarat Karinchai, Hideki Yoshida, Masamitsu Yamaguchi, Panuwan Chantawannakul

**Affiliations:** 1 Graduate School, Chiang Mai University, A. Meung, Chiang Mai, 50200 Thailand; 2 Bee Protection laboratory, Department of Biology, Faculty of Science, Chiang Mai University, A. Meung, Chiang Mai, 50200 Thailand; 3 Department of Applied Biology, Kyoto Institute of Technology, Matsugasaki, Sakyo-ku, Kyoto, 606-8585 Japan; 4 Department of Pharmacology, Faculty of Medicine, Chiang Mai University, A. Meung, Chiang Mai, 50200 Thailand; 5 Department of Biochemistry, Faculty of Medicine, Chiang Mai University, A. Meung, Chiang Mai, 50200 Thailand; 6 The Center for Advanced Insect Research, Kyoto Institute of Technology, Matsugasaki, Sakyo-ku, Kyoto, 606-8585 Japan; 7 Environmental Science Research Center, Faculty of Science, Chiang Mai University, A. Meung, Chiang Mai, 50200 Thailand

**Keywords:** Coffee honey, Edible insect, Honeybee product, Neuroprotective agent, *Ubiquilin*

## Abstract

**Background:**

Mutations in the human *Ubiquilin 2* gene are associated with neurodegenerative diseases such as amyotrophic lateral sclerosis (ALS) with or without frontotemporal dementia (FTD), the fatal neurodegenerative disease that progressively affected neuronal cells in both brain and spinal cord. There is currently no effective therapy for these diseases. Over the last decade, researchers have focused on the potential use of natural products especially in neurodegenerative studies. Insect products have been used as traditional medicines, however, scientific information is still lacking. Fruit fly is recently used as a model organism to investigate degenerative diseases related to the nervous system because it has a short life span and produces a large number of offspring.

**Methods:**

The present study investigated the effects of honeybee products and edible insect powders on the locomotive and learning abilities, neuromuscular junctions (NMJs) structure, and reactive oxygen species (ROS) in larval brains of *Ubiquilin-* knockdown *Drosophila.*

**Results:**

*dUbqn* knockdown flies showed defects in locomotive and learning abilities accompanied with structural defects in NMJs. The results obtained revealed that the recovery of locomotive defects was significantly greater in *dUbqn* knockdown flies fed with coffee honey from *Apis cerana* (1% v/v) or *Apis dorsata* melittin (0.5 μg/ml) or wasp powder (2 mg/ml) than that of in untreated *dUbqn* knockdown flies. Furthermore, *dUbqn* knockdown flies fed with coffee honey showed the partial rescue of structural defects in NMJs, improved learning ability, and reduced the accumulation of ROS caused by *dUbqn* depletion in the brain over the untreated group.

**Conclusion:**

These results suggest that coffee honey from *Apis cerana* contains a neuroprotective agent that will contribute to the development of a novel treatment for ALS/FTD.

## Background

Entomophagy or eating insects as food is well known in many cultures worldwide. Edible insects are a promising, healthy, and sustainable source of high quality proteins, good amino acids, and various fatty acids and contain high contents of a number of micronutrients, such as the minerals copper, iron, magnesium, manganese, phosphorous, selenium, and zinc as well as vitamins [1, 2]. Besides insects, insect-derived products, such as honeybee products, are well known and have been used since ancient times [3–5]. Honeybees produce honey, royal jelly, propolis, pollen, honeybee wax, and venom, which are potentially beneficial to humans due to their bioactive compounds [6–9]. Honeybee products are also regarded as a potential source of natural antioxidants [10]. The antioxidant activities of honeybee products are linked to their polyphenol compounds [9, 11, 12]. Phenolic and flavonoid compounds are the most important group of antioxidant substances present in honeybee products. The diversity of the phenolic content and antioxidant capacity depends on the processing, handling, types of plants, honeybees species, climatic conditions, geographical region and storage of honeybee products [13–16].

Insects and insect-derived products have been used in traditional medicine, a practice known as entomotherapy [17, 18]. In Chinese traditional medicine, insects and their products such as silkworms (*Bombyx mori* L., 1758) have been applied for more than 3,000 years [19]. The larvae of certain flies have been recognized as an effective treatment for infected wound healing [20]. In addition, house crickets (*Acheta domesticus* L., 1758) have been used to cure scabies, asthma, eczema, earache, rheumatism, urine retention, urinary incontinence and ophthalmological problems in Latin American [21]. Honeybees and their products (e.g. honey, royal jelly, and venom) have been recorded in traditional medicine or a so-called “apitherapy” to treat/prevent various diseases [4]. As known, honey contains a number of phenols, the important honey phenols including flavonols (quercetin, kaempferol, galangin, fisetin, and myricetin), flavones (apigenin, acacetin, chrysin, luteolin, genkwanin, wogonin, and tricetin), phenolic acids (caffeic acids), and flavanones (hesperidin) [11, 12, 16]. Of these compounds, the flavonoid quercetin has been reported to enhance the apoptotic abilities of anti-CD95 and recombinant tumour necrosis factor-related apoptosis-inducing ligand (rTRAIL) in the treatment of acute lymphocytic leukemia [22]. Honey is also used for the cure of many human diseases skin disorders, gastrointestinal disorders, neurological diseases, cardiovascular disorders, and diseases of the intestine [23–28]. Royal jelly, a larval food of honeybees, is also used to treat a wide variety of diseases, such as asthma, anorexia, gastrointestinal ulcers, arteriosclerosis, anemia, hypo- or hypertension, anti-cancer, wound healing, and neurasthenia or inhibit sexual libido [29–33]. Moreover, the venom of the honeybee has been employed to reduce the symptoms of and ameliorate inflammatory and autoimmune diseases, such as multiple sclerosis, arthritis, rheumatism, chronic pain, asthma, neurological diseases, and dermatological conditions [34–40]. The final apitherapy product, honeybee pollen, is considered a health food with a wide range of therapeutic properties such as antifungal, antimicrobial, antiviral, anti-inflammatory, immunostimulating, and the burn wound healing [41–44]. However, little is known about the in vivo effects of insects and insect-derived products which have long been consumed in a daily life especially on neurodegenerative diseases.

Amyotrophic lateral sclerosis (ALS) with or without frontotemporal dementia (FTD) as one of neurodegenerative disease that affects the motor neurons connecting the brain and spinal cord, leading to eventual paralysis and death, and a mean survival of 2–5 years of life after diagnosis [45]. Mutations in the gene encoding ubiquitin chaperone *Ubiquilin 2* (*UBQLN2*) have been associated with the early onset of neurodegenerative diseases, including ALS/FTD [46–48]. Mutations in *UBQLN2* have also been identified as a cause of the X-linked forms of ALS/FTD [49]. Furthermore, mutations in *UBQLN2* have recently been detected in familial cases of amyotrophic lateral sclerosis (FALS) and frontotemporal lobar dementia (FTLD) [50]. Recent studies demonstrated that some ALS-related *UBQLN2* mutants exhibited defects in ubiquitin-binding abilities that resulted in a failure to deliver ubiquitinated substrates to proteasomes for degradation [46].

The fruit fly *Drosophila melanogaster* is the most commonly used experimental model organism. The advantage of using *D. melanogaster* is its genetic similarity to humans because approximately 75% of human disease-associated genes have related sequences in *D. melanogaster* [51, 52]. Furthermore, *Drosophila* is emerging as a useful model organism for advancing our understanding of the pathogenic mechanisms of *UBQLNs*-associated neurological diseases because of its simplicity; it carries a single orthologue of human *UBQLNs*, called *Drosophila Ubiquilin* (*dUbqn*), which shows high similarity to both human *UBQLN1* (50% identity) and *UBQLN2* (48% identity), respectively, and also carries conserved functional domains [53]. Therefore, *Drosophila* larvae have widely used as a model to study human neurodegenerative diseases including ALS/FLTD based on the following three criteria [54]. Firstly, locomotive ability can be quantitatively evaluated by the crawling assay in larvae. The progressive impairment in locomotive ability of the ALS/FLTD fly models has been characterized through crawling assay at larval stage [54]. Secondly, the *Drosophila* larval neuromuscular junctions (NMJs) allow easy and quantitative analyses of structural connections between the motor neuron and the muscle [55]. It is also reported that locomotive ability defects of larvae are frequently accompanied by synapse structural defects at NMJs [54]. Thirdly, learning ability can be readily examined at larval stage of *dUbqn* knockdown flies, which exhibited locomotive dysfunctions and cognitive impairments in combination with severe structural defects in NMJs and mushroom bodies [54].

Currently, there are only two drugs (riluzole and edaravone) approved by U.S. food and drug administration (FDA) bringing a new hope for ALS patients. Unfortunately, the long-term effects and side effects of these drugs are still insufficiently investigated [56]. Moreover, an accessibility of new drugs is still being a great burden in some developing countries. Recently, the screening of natural products is gaining an attention, because the products can be generally used in daily life and are considered lack of toxicity. Especially in the field of neurodegeneration, many of herbs and insect products such as curcumin and melittin, have been found to be effective to delay or prevent the disease progression in cell and animal models [57, 58]. It is therefore of our interest to investigate whether honeybee products and edible insect powders have potential to rescue the neurological defects caused by Ubiquilin depletion using *Drosophila* as a model organism.

## Methods

### Honeybee products and edible insect powders preparation

We used 15 samples of insects and insect-derived products in this present study. Edible insects and honeybee products that we used in this study were obtained from Northern Thailand. These edible insects and honeybee products exhibited high nutrition values and also pharmacological properties such as antioxidant, antimicrobial, and anti-inflammatory activities [9, 16, 59].

i. Honeybee products. Honeybee product samples and their honeybee producers were *Apis mellifera* (longan honey, tea pollen, and fresh royal jelly) and *Apis cerana* (coffee honey). Longan honey, coffee honey, and tea pollen samples were purchased from local suppliers in Chiang Mai, Thailand, and kept in a lightproof container at 23–25 °C until used. Fresh royal jelly samples were kept at − 20 °C until used. Melittin peptides (purity > 95%) from different species of *Apis* in Thailand (*A. cerana*, *A. dorsata*, and *A. florea*) were synthetized from Bio Basic Canada Inc. (Lot: 20180726, Markham, Ontario, Canada). All samples were kept at − 20 °C until used.
ii. Edible insects. *Bombyx mori* (silkworm (larvae)), *Gryllus bimaculatus* (crickets (adult)), *Lethocerus indicus* (giant water bug (adult)), *A. mellifera* (European honeybee queen (larvae)), *Vespa affinis* (wasp (pupae)), *Pompania* sp. (cicada (pupae)), *Omphisa fuscidentalis* (bamboo borer (larvae)), and *Carebara castanea* (subterranean ant (adult)) were purchased in March – April 2018 from a local supplier in the northern area of Thailand. All samples were certified and authorized for human consumption. Samples were kept at − 20 °C until used for experiments.
iii. Edible insect powders and royal jelly powder preparation. Freeze-dried fresh royal jelly (FDRJ), samples were prepared from fresh royal jelly and stored at − 20 °C. Pooled frozen samples were thawed and filtered. After filtration, liquid royal jelly was converted into a powder using a vacuum dryer. Freeze-dried royal jelly powder (FDRJ) was obtained. In each species of edible insects, individuals were pooled and homogenized with a blender. The mixture was filtered with a 100-mesh filter, followed by Whatman No.1 filter paper. After filtration, samples were freeze-dried. Freeze-dried edible insects and FDRJ were packed in a lightproof container and stored at 4 °C until used in the present study.

### Fly stocks and maintenance

All flies were cultured at 25 °C with standard *Drosophila* food containing agar (0.65% w/v), glucose (10% w/v), dry yeast (4% w/v), corn flour (5% w/v), and rice powder (3% w/v). Fly lines carrying *w*; *UAS-dUbqnIR*_*107–494*_; + (ID 106050) were obtained from the Vienna *Drosophila* Resource Center (VDRC; Vienna, Austria). Fly lines carrying *w*; *+*; *elav-GAL4* (RRID: BDSC_8760) and *w*; *UAS-GFPIR*;+ (RRID: BDSC_41550) were obtained from the Bloomington *Drosophila* Stock Center (BDSC; Bloomington, Indiana, USA).

### Drosophila culture and crosses

The UAS/GAL4 system for gene silencing was used to knockdown *dUbqn* in this study. Briefly, virgin females *w*;*+*; *elav-GAL4* (RRID: BDSC_8760) which harbored the construct for pan-neuronal GAL4 expression, were crossed with *w*/Y; *UAS-dUbqnIR*_*107–494*_;+ (ID 106050) males which carried the construct of transgenic expression of RNAi against *dUbqn* at the region corresponding to the N-terminal dUbqn domain from aa position: aa 107 to aa 494 [54], inside the vials containing instant *Drosophila* medium blue (Carolina Biological Supply Company, Burlington, North Carolina, USA) with two concentrations of test samples mixed in (Supplementary Table S1 and S2) and maintained at 28 °C. The progenies (F1) which were knocked down of pan-neuronal *dUbqn,* were used to perform all experiments. The knockdown efficiency was confirmed by mRNA expression using quantitative RT-PCR. Forty of third instar larval whole brains were used for each genotype (control and *dUbqn* knockdown). Total RNA was extracted using the RNeasy Lipid Tissue Mini-Kit (Qiagen, Hilden, Germany) and following a DNase treatment (DNase I, Roche, Mannheim, Germany), 500 ng of total RNA was reverse-transcribed to cDNA using the Primescript RT reagent kit (TaKaRa, Shiga, Japan) according to the manufacturer’s instructions. Quantitative RT-PCR was performed in triplicate for each single extraction with the SYBR Green Master Mix: SYBR Premix Ex Taq II (Tli RNase H Plus, TaKaRa, Shiga, Japan) with the CFX96 Touch™ Real-Time PCR Detection System (Bio-Rad, USA) using the following specific primer pairs: *dUbqn* (F *dUbqn* 5′- AACGCCCTTTGGCCTCAAT-3′, R *dUbqn* 5′-CAC CATCGGGTTGTCCATCA-3′); *RpL32* (F *RpL32* 5′-AGATCGTGAAGAAGCGCACC-3′, R *RpL32* 5′-CGATCCGTAACCGATGTTGG-3′).

Samples were run in triplicate, and data were analyzed with a standard curve-based method calculated with CFX Manager™ software (Bio-Rad). The specificity of primers was tested with melt curves created by CFX Manager™ software. *RpL32* was used as an internal control [54]. The *dUbqn* knockdown larvae without treatment were considered as untreated flies. All experiments, we used flies carrying *w*; *UAS-GFPIR/+;elav-GAL4/+* as control flies. The uptake of food was confirmed by inspecting a blue color in the larval intestine. Third-instar larvae were selected by size and wandering behavior.

### Crawling assay

A crawling assay was performed as described previously [60] with some modifications. Flies carrying *w*; *UAS-dUbqnIR*_*107–494*_*/+;elav-GAL4* were cultured at 28 °C with instant *Drosophila* medium containing two concentrations of insect and insect-derived product samples (Supplementary Table S1 and S2). Flies carrying *w*; *UAS-GFPIR/+;elav-GAL4/+*, (control flies) and *w*; *UAS-dUbqnIR*_*107–494*_*/+;elav-GAL4* (untreated flies) were cultured at 28 °C with instant *Drosophila* medium. Larvae at the third instar wandering stage were collected and washed with phosphate-buffered saline (PBS) to remove food traces. Larvae were then transferred into a 15-cm Petri dish containing 2% (w/v) agarose at a density of two larvae per plate. A 1-min video was acquired with a digital camera. Recorded videos were then converted to the AVI type using a MOV to AVI converter (Pazera Jacek, Poland) and analyzed by ImageJ software with the wrMTrck plug-in to track larval movement and draw motion paths.

### Larval learning assay

Third instar larvae earlier than the wandering stage were trained and tested for odor-taste learning performance according to previously described protocols [54], in which experiments were conducted on assay plates. Based on the results of locomotive abilities, we selected three treatments (coffee honey (1% v/v), *A. dorsata* melittin (0.5 μg/ml), and wasp powder (2 mg/ml)) for larval learning ability experiment. Flies carrying *w*; *UAS-dUbqnIR*_*107–494*_*/+;elav-GAL4* were cultured at 28 °C with instant *Drosophila* media containing different tested samples (coffee honey (1% v/v), *A. dorsata* melittin (0.5 μg/ml), wasp powder (2 mg/ml)). Flies carrying *w*; *UAS-GFPIR/+;elav-GAL4/+*, (control flies) and *w*; *UAS-dUbqnIR*_*107–494*_*/+;elav-GAL4* (untreated flies) were cultured at 28 °C with instant *Drosophila* medium. The learning assay is based on two phases. A group of larvae was firstly exposed to n-amyl acetate (AM; Merck, Darmstadt, Germany) in the presence of a reward (2 M sucrose, SUC) and then 1-octanol (OCT; Sigma, Steinheim, Germany) in the absence of SUC. This training was defined as AM+/OCT, with “+” indicating the reward. Reciprocal training was then carried out. The group of larvae was sequentially exposed to OCT in the presence of SUC and then AM in the absence of SUC. This training is defined as OCT+/AM. To allow flies to make an association between AM or OCT and the reward (SUC), five minutes of each training were performed and repeated three times. Each odorant used in the experiments (OCT and AM) were prepared by loading ten μl onto a 200 μl-PCR tube (perforated lid) with 7 holes and placed on the opposite site inside the Petri dish. Undiluted OCT was used, while AM was applied at a 1:50 dilution with liquid paraffin. Eighteen larvae for each corresponding training (AM+/OCT and OCT+/AM, respectively) were then divided into three groups. Each group of larvae (*n* = 6) was tested by exposure to AM and OCT, which were deposited on the opposite ends of each test plate in the absence of SUC, for four minutes. Memory ability was evaluated by comparing the preference for the reward odorant against that for the no reward one. The preference indexes were used to calculate a learning index (LI).

### Visualization of NMJs

Flies carrying *w*; *UAS-dUbqnIR*_*107–494*_*/+;elav-GAL4* were cultured at 28 °C with instant *Drosophila* media containing different tested samples (coffee honey (1% v/v), *A. dorsata* melittin (0.5 μg/ml), wasp powder (2 mg/ml)). We selected three treatments according to the locomotive abilities results. Flies carrying *w*; *UAS-GFPIR/+;elav-GAL4/+*, (control flies) and *w*; *UAS-dUbqnIR*_*107–494*_*/+;elav-GAL4* (untreated flies) were cultured at 28 °C with instant *Drosophila* medium. Third instar larvae (10 larvae/group) were dissected in HL3 saline and fixed in 4% paraformaldehyde in PBS at 25 °C for 30 min [61] NMJs were stained as previously describe [62]. The blocking buffer contained 2% bovine serum albumin and 0.1% Triton X-100 in PBS buffer was used at 25 °C for 30 min. Fluorescein isothiocyanate (FITC)-conjugated goat anti-horseradish peroxidase (HRP) IgG (1:1000 dilution, MP Biochemicals) was applied as the detection antibody and then probed with mouse monoclonal anti-Disc large (Dlg) (1:300 dilution, DSHB). After being washed with PBS buffer containing 0.3% Triton X-100, samples were incubated with secondary antibodies labeled with Alexa 594 (1:400 dilution) at 25 °C for 3 h. After extensive washing with PBS containing 0.3% Triton X-100, stained samples were then mounted with ProLong Diamond (Invitrogen, USA) and MN 4 (Ib) on muscle number 4 belonging to abdominal segment 3 was observed and quantified under a confocal laser-scanning microscope (Fluoview FV10i, Olympus, Tokyo, Japan). Images were taken with a super resolution microscope (N-SIM, Nikon, Japan).

### Measurement of reactive oxygen species (ROS) levels

Flies carrying *w*; *UAS-dUbqnIR*_*107–494*_*/+;elav-GAL4* were cultured at 28 °C with instant *Drosophila* medium containing coffee honey (1% v/v). We selected the coffee honey (1% v/v) treatment base our study on learning abilities and NMJs structure. Flies carrying *w*; *UAS-GFPIR/+;elav-GAL4/+*, (control flies) and *w*; *UAS-dUbqnIR*_*107–494*_*/+;elav-GAL4* (untreated flies) were cultured at 28 °C with instant *Drosophila* medium. The brains from third instar larvae of control, untreated, and coffee honey (1% v/v)-fed groups (20 larvae/group) were placed in 1× PBS (17.5 mM NaCl, 8.41 mM Na_2_HPO_4_, and 1.86 mM NaH_2_PO_4_, pH 7.4). Larval brains from the control, untreated, and treated groups (20 larvae/group) were homogenized in cold 1× PBS (pH 7.4) followed by centrifugation at 12,000×*g* for 10 min. Total ROS levels were measured in homogenates of the larval brains from control, untreated, and coffee honey (1% v/v)-fed groups using 2′,7′-dihydrofluorescein diacetate (H_2_DCFDA; Invitrogen) following the method reported previously [63] with minor modifications. Briefly, in brain homogenates, the dye was added at a final concentration of 10 μM and incubated at 24 ± 1 °C for 1 h in the dark. The mixture was then placed on a microplate reader (Molecular Devices LLC, California, USA) for fluorescence quantification at an excitation/emission wavelength of 495/519 nm. The mean fluorescence intensity was used to estimate ROS levels in each sample. Three samples from each group were analyzed in triplicate.

### Statistical analysis

Statistical analyses were performed with GraphPad Prism version 6.02. The Mann-Whitney U test was used to test for significant differences between groups of independent data, while comparisons between groups were performed by the Kruskal-Wallis test or a two-way ANOVA followed by Dunnett’s multiple comparison analysis. All data were shown as means ± SEM. *P* values of < 0.05 were considered significant for all.

## Results

### Honeybee products and edible insect powders feeding improved the locomotive activity of *dUbqn* knockdown larvae

**Fig. 1:**
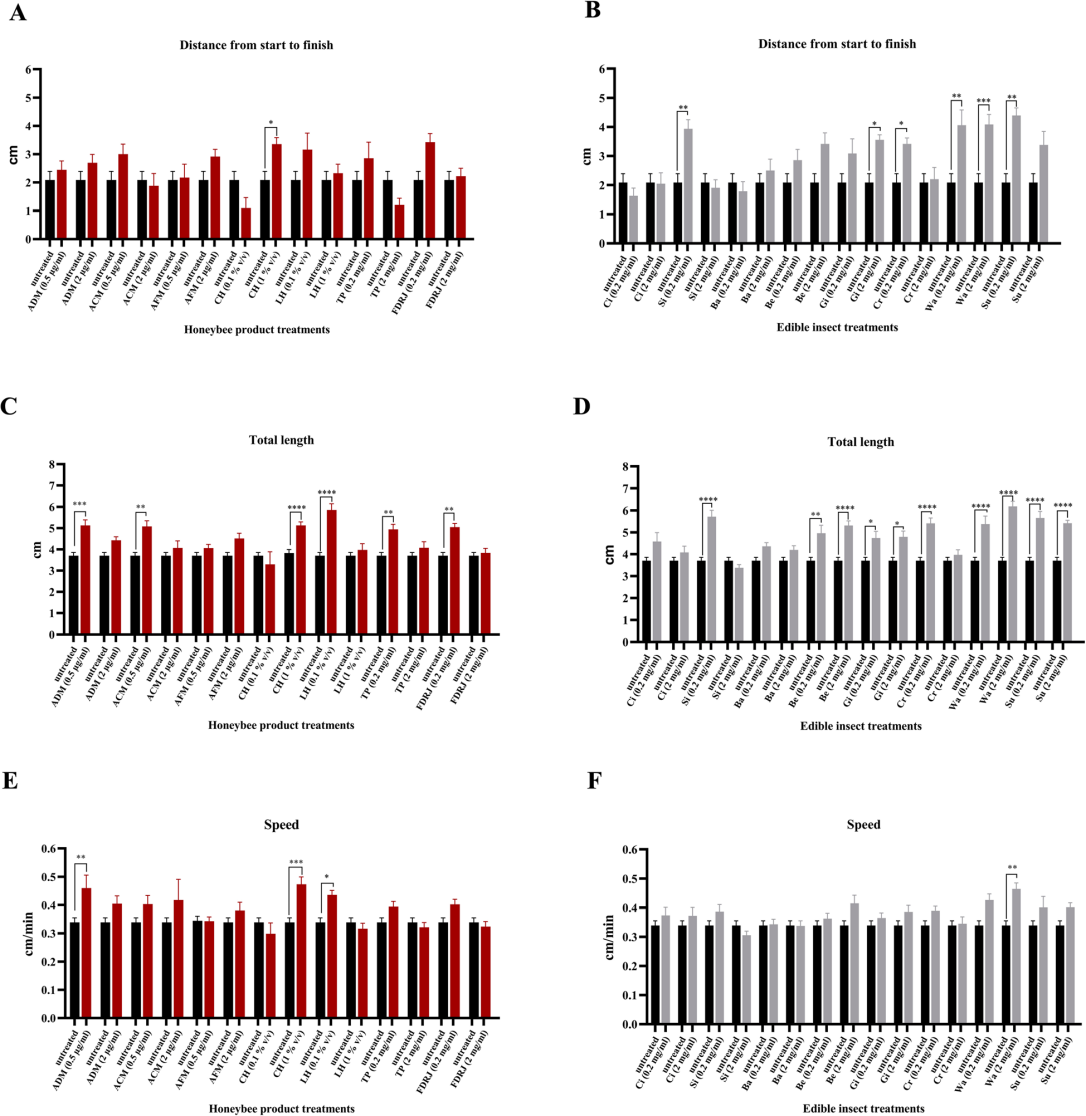
Effects of honeybee products and edible insect powders on locomotive abilities of pan neuron-specific *dUbqn* knockdown (*w*; *UAS-dUbqnIR*_*107–494*_*/+;elav-GAL4*). The distance (**a**, **b**), length (**c**, **d**), and speed (**e**, **f**) of *dUbqn* knockdown larvae covered in one minute in comparison between untreated and treated conditions. The results represent mean ± SEM. * *p* < 0.05, ** *p* < 0.01, *** *p* < 0.001, **** *p* < 0.0001 (*n* = 20 larvae per group). ADM: *Apis dorsata* melittin; ACM: *Apis cerana* melittin; AFM: *Apis florea* melittin; CH: coffee honey; LH: longan honey; TP: tea pollen; FDRJ: freeze-dried fresh royal jelly; Ci: cicada powder; Si: silkworm powder; Ba: bamboo borer powder; Be: honeybee larva powder; Gi: giant water bug power; Cr: crickets powder; Wa: wasp power; Su: subterranean ants powder

We examined the effects of insects and insect-derived products on the locomotive abilities of pan neuron-specific *dUbqn* knockdown larvae. To evaluate knockdown efficiency of *dUbqn* in the RNAi line, a transcriptional gene analysis was performed using RNA extracts from the third instar larval brains to measure the abundance of *dUbqn* transcripts with respect to an internal control, *RpL32.* The *dUbqn* knockdown larvae showed 38% reduction in *dUbqn* transcript levels from the control (Supplementary figure S1). The *Drosophila* strain using in the present study was previously confirmed for no off-target effects [54]. Using ImageJ, crawling parameters were quantified, including distance (cm, distance from start to finish), speed (cm/min), and length (cm, total path length). We measured the distance covered in 1 minute by the treated larvae and compared it with those of untreated larvae (Fig. 1). The depiction of the larval path showed that untreated *dUbqn* knockdown larvae were confused and had a shorter crawling distance, whereas larvae treated with the samples described below moved linearly and covered a greater distance (Fig. 1a and b). The crawling distances of larvae treated with coffee honey (1% v/v), silkworm powder (0.2 mg/ml), giant water bug powder (2 mg/ml), cricket powder (0.2 mg/ml), wasp powder (0.2, 2 mg/ml), and subterranean ant powder (0.2 mg/ml) were significantly longer than that of untreated larvae (*p* = 0.0151 (*n* = 20), *p* = 0.0119 (*n* = 20), *p* = 0.0308 (*n* = 20), *p* = 0.0281 (*n* = 20), *p* = 0.0025 (*n* = 20), *p* = 0.0003 (*n* = 20), and *p* = 0.0015 (*n* = 20), respectively) (Fig. 1a and b, *n* = 20). In contrast, the treatment of larvae with *A. dorsata* melittin (0.5 and 2 μg/ml), *A. cerana* melittin (0.5 and 2 μg/ml), *A. florea* melittin (0.5 and 2 μg/ml), coffee honey (0.1% v/v), longan honey (0.1 and 1% v/v), tea pollen (0.2 and 2 mg/ml), FDRJ (0.2 and 2 mg/ml), cicada powder (0.2 and 2 mg/ml), silkworm powder (2 mg/ml), bamboo borer powder (0.2 and 2 mg/ml), honeybee larvae powder (0.2 and 2 mg/ml), giant water bug powder (0.2 mg/ml), cricket powder (2 mg/ml), and subterranean ant powder (2 mg/ml) did not significantly affect crawling distances.

The crawling speeds of pan neuron-specific *dUbqn* knockdown larvae fed with coffee honey (1% v/v), longan honey (0.1% v/v), *A. dorsata* melittin (0.5 μg/ml), and wasp powder (2 mg/ml) were significantly faster than that of untreated larvae (*p* = 0.0002, (*n* = 20), *p* = 0.0236 (*n* = 20), *p* = 0.0096 (*n* = 20), and *p* = 0.046 (*n* = 20), respectively) (Fig. 1e and f, n = 20). In contrast, the treatment of larvae with *A. dorsata* melittin (2 μg/ml), *A. cerana* melittin (0.5 and 2 μg/ml), *A. florea* melittin (0.5 and 2 μg/ml), coffee honey (0.1% v/v), longan honey (1% v/v), tea pollen (0.2 and 2 mg/ml), FDRJ (0.2, and 2 mg/ml), cicada powder (0.2 and 2 mg/ml), bamboo borer powder (0.2 and 2 mg/ml), silkworm powder (0.2 and 2 mg/ml), giant water bug powder (0.2 and 2 mg/ml), cricket powder (0.2 and 2 mg/ml), wasp powder (0.2 mg/ml), subterranean ant powder (0.2 and 2 mg/ml), and honeybee larvae powder (0.2 and 2 mg/ml) did not significantly increase crawling speeds. Collectively, these results indicate that three samples, coffee honey (1% v/v), *A. dorsata* melittin (0.5 μg/ml), and wasp powder (2 mg/ml), are good candidates for improving the defects induced by the pan neuron-specific *dUbqn* knockdown in *D. melanogaster*.

### Coffee honey feeding improved learning ability of *dUbqn* knockdown larvae

**Fig. 2:**
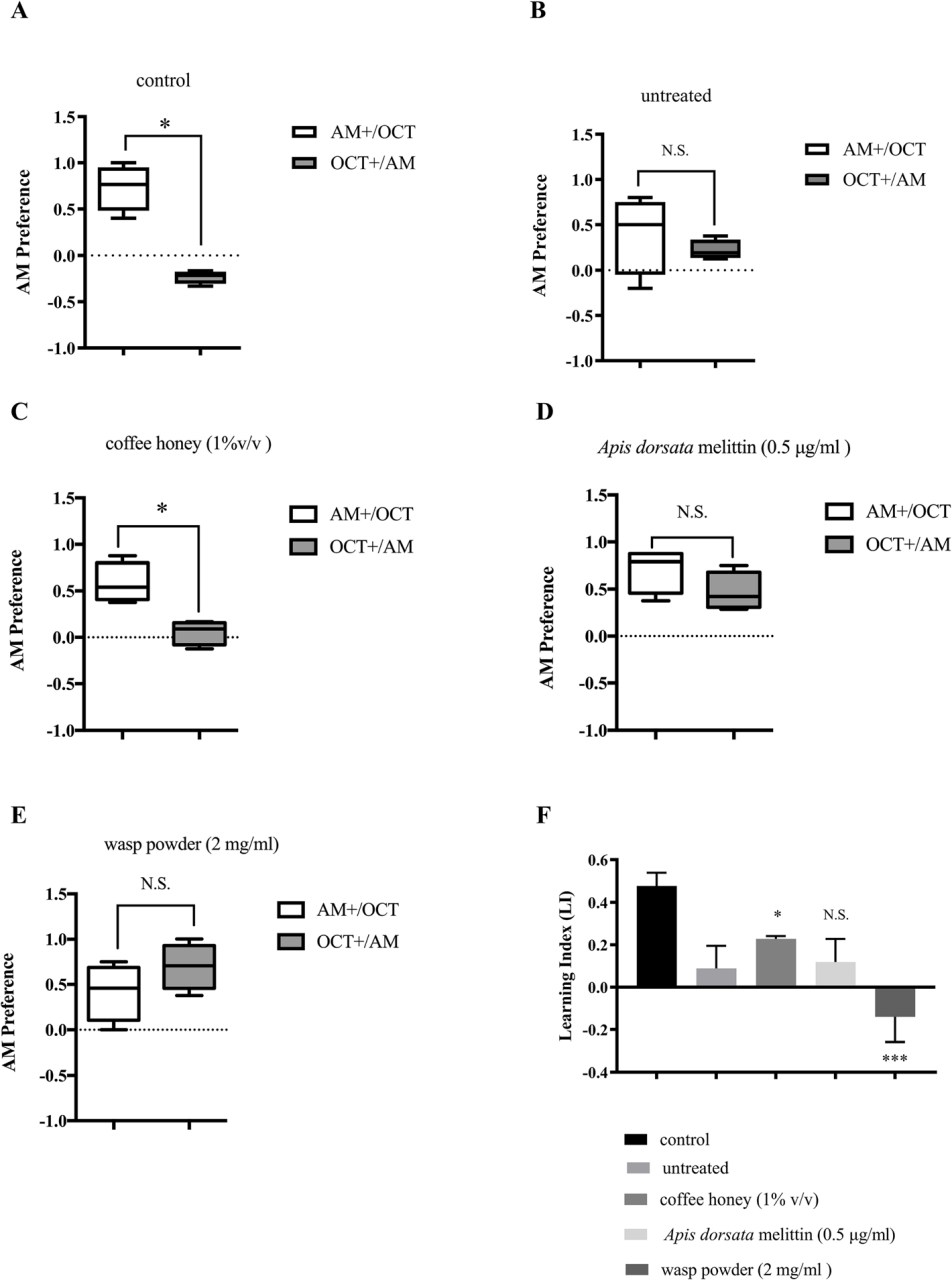
Effects of honeybee products and edible insect powders on learning abilities of pan neuron-specific *dUbqn* knockdown larvae. The larvae were sequentially exposed to n-amyl acetate (AM) in the presence of reward and then 1-octanol (OCT) in the absence of reward (AM+/OCT). The reciprocal training the larvae was sequentially exposed to OCT in the presence of reward and AM in the absence of reward (OCT+/AM). After training, larvae were tested by exposure to AM and then OCT in the absence of reward to test their preference. Larval AM preference score was shown in (**a-e**) (score = 1 means all preferred AM; score = − 1 means all preferred OCT). The preference score of control larvae (*w*; *UAS-GFPIR/+;elav-GAL4/+*) and *dUbqn* knockdown larvae (*w*; *UAS-dUbqnIR*_*107–494*_*/+;elav-GAL4*) without treatment was shown in (**a**, **b**) whereas the preference score of *dUbqn* knockdown larvae treated with coffee honey (1% v/v), *A. dorsata* melittin (0.5 μg/ml), and wasp powder (2 mg/ml) was shown in (**c**, **d**, **e**). Box-and-whisker plots represent the minimum, first quartile, median, third quartile, and maximum. Normalized learning index of all groups was shown in (**f**). The higher LI means the higher ability of larvae to learn and perform the conditional tasks. The results represent mean ± SEM. * *p* < 0.05, N.S. not significant. (*n* = 3 of 6 larvae per group)

Based on their effects on locomotive ability, three treatments (coffee honey (1% v/v), *A. dorsata* melittin (0.5 μg/ml), and wasp powder (2 mg/ml)) were selected for further study on their effects on learning/memory abilities. We compared the learning ability of the treated groups with that of the untreated group. To investigate the effects of the three samples on the role of *dUbqn* in complex neuronal functions, we performed a *Drosophila* larval odor-taste learning assay. Using this approach, we tested larvae carrying *w*; *UAS-GFPIR/+;elav-GAL4/+* (control flies) and *w*; *UAS-dUbqnIR*_*107–494*_*/+;elav-GAL4/+* (pan neuron-specific *dUbqn* knockdown flies) (Fig. 2). In the control larvae, the preference for the reward odorant was greater than that to the no reward odorant in accordance with training. The result displays their abilities to make the correct association between an odorant and reward (Fig. 2a). In *dUbqn* knockdown larvae (untreated flies), the AM preference index was not significantly higher in AM+/OCT than in OCT+/AM (Fig. 2b). These results indicate that *dUbqn* knockdown larvae did not make an association between the odorant and reward. Therefore, it clearly showed that the depletion of *dUbqn* resulted in a reduction in learning abilities in *dUbqn* knockdown larvae (Fig. 2f).

The *dUbqn* knockdown larvae treated with coffee honey (1% v/v) showed a greater preference to the reward odorant than to the no reward odorant in accordance with training (Fig. 2c). However, the treatment with *A. dorsata* melittin (0.5 μg/ml) and wasp powder (2 mg/ml) did not improve the ability of *dUbqn* knockdown larvae to make an association between the odorant and reward (Fig. 2d and e). LI clearly showed that among these three treatments (coffee honey (1% v/v), *A. dorsata* melittin (0.5 μg/ml), and wasp powder (2 mg/ml)), only the coffee honey (1% v/v)-treated group showed significantly better learning ability (*p* < 0.05) in learning performance (learning scores around 0.22) than the untreated group (Fig. 2f). These results indicate that coffee honey is solely effective for recovering the learning defects induced by the *dUbqn* knockdown.

### Coffee honey feeding rescued the synapse structural defects at NMJs induced by the *dUbqn* knockdown

**Fig. 3:**
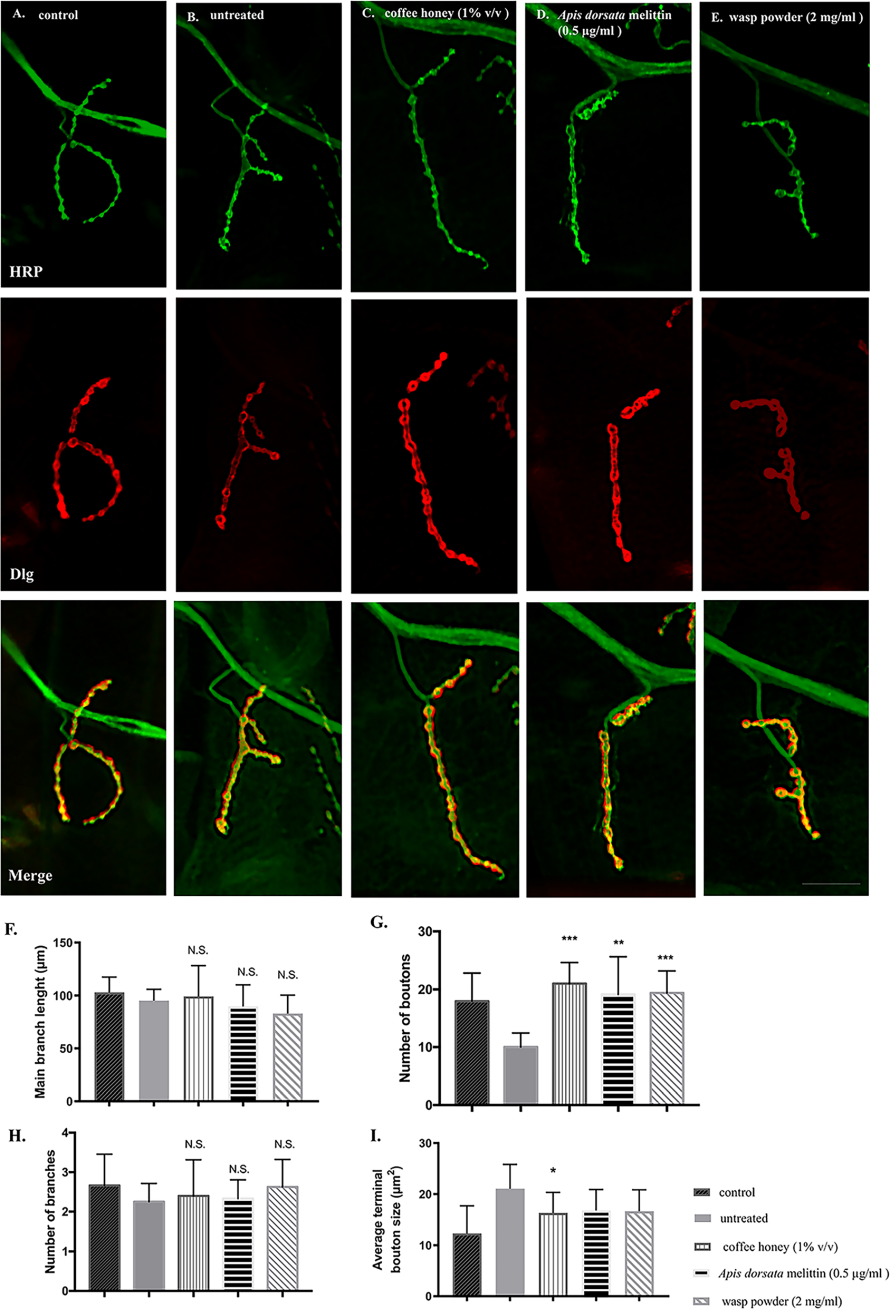
Effects of honeybee products and edible insect powders on synaptic structures. The synaptic structures of control larvae (*w*; *UAS-GFPIR/+;elav-GAL4/+*) (**a**), pan neuron-specific *dUbqn* knockdown larvae (*w*; *UAS-dUbqnIR*_*107–494*_*/+;elav-GAL4*) without treatment (**b**), with coffee honey treatment (**c**), *Apis dorsata* melittin treatment (**d**), and wasp powder treatment (**e**) were shown. HRP and Dlg which are pre- and post-synaptic markers were observed in green and red signals, respectively. The quantification of the main branch length (**f**), number of boutons (**g**), number of branches (**h**), and average terminal boutons size (**i**) was performed by ImageJ. Quantification results represent the mean ± SEM. * *p* < 0.05, ** *p* < 0.01, *** *p* < 0.001, N.S. not significant. Scale bar is 30 μm. (*n* = 10 larvae per group)

We examined whether the three treatments (coffee honey (1% v/v), *A. dorsata* melittin (0.5 μg/ml), and wasp powder (2 mg/ml)) have abilities to rescue the synapse structural defects at NMJs. When we compared the structure of NMJs at larval segment A3 in muscle number 4 in third instar larvae with the pan neuron-specific knockdown of *dUbqn* (*w; UAS-dUbqnIR107–494/+;elav-GAL4/+*) with a control flies (*w*; *UAS-GFPIR/+;elav-GAL4/+*), the NMJ structure of *dUbqn* knockdown larvae (untreated flies) showed a significantly shortened main branch length (90.3 ± 3.8 μm, *dUbqn* knockdown larvae vs 106.6 ± 5.6 μm, control larvae, *p* < 0.05, Fig. 3f) and a decrease in the number of boutons (10.8 ± 0.8, *dUbqn* knockdown larvae vs 17.9 ± 1.1, control larvae, *p* < 0.05, Fig. 3g). A significantly larger terminal bouton size was also observed in *dUbqn* knockdown larvae (20.1 ± 1.7 μm^2^, *dUbqn* knockdown larvae vs 12.3 ± 1.2 μm^2^, control larvae, *p* < 0.05, Fig. 3i).

The reduced number of boutons in *dUbqn* knockdown larvae was significantly rescued by the three treatments (Fig. 3g). Only coffee honey (1% v/v) rescued the enlarged terminal bouton size induced by the *dUbqn* knockdown (Fig. 3i). In contrast, the increase observed in NMJ arbor branch numbers in *dUbqn* knockdown flies was not significantly restored by these three treatments. Thus, coffee honey appeared to be the most effective at rescuing the synapse structural defects at NMJs induced by the *dUbqn* knockdown.

### Coffee honey feeding reduced the accumulation of ROS in *dUbqn* knockdown flies

**Fig. 4:**
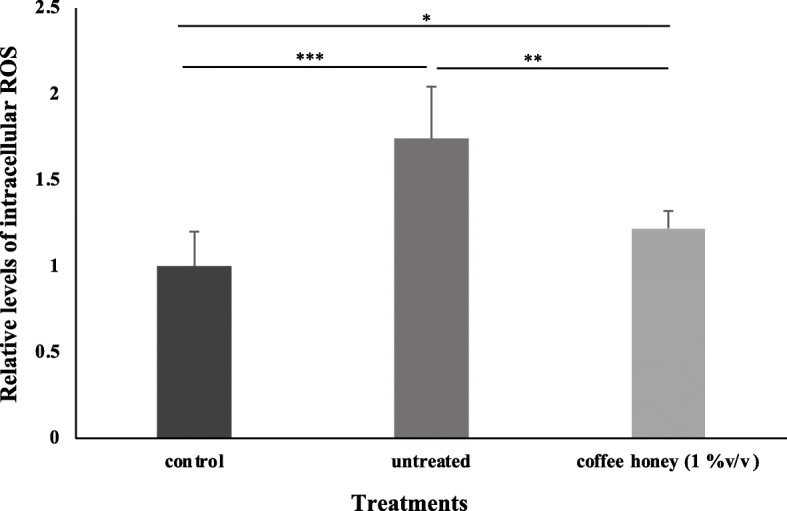
Reactive oxygen species (ROS) generation in response to coffee honey. The ROS levels of the control larvae (*w*; *UAS-GFPIR/+;elav-GAL4/+*), *dUbqn* knockdown larvae (*w*; *UAS-dUbqnIR*_*107–494*_*/+;elav-GAL4*) without treatment and with coffee honey treatment were measured using fluorescence emitted by dichlorofluorescein (DCF). Quantification results represent mean ± SEM. * *p* < 0.05, ** *p* < 0.01, *** *p* < 0.001. (*n* = 3 of 20 larvae per group)

Total ROS levels were measured in the homogenates of third instar larval brains. To elucidate the association between the knockdown of *dUbqn* and oxidative stress, we measured ROS levels in the third instar larval brains of *dUbqn* knockdown flies. The results displayed that ROS levels in third instar larval brains were 1.7-fold higher in *dUbqn* knockdown larvae (untreated flies) than in control larvae (Fig. 4). These results suggest that the knockdown of *dUbqn* increased ROS levels in *Drosophila* larval brains. It is interesting to note that the ROS levels in third instar larval brains were 0.5-fold lower in *dUbqn* knockdown larvae treated with coffee honey (1% v/v) than in untreated *dUbqn* knockdown larvae (Fig. 4). These results clearly demonstrated that the knockdown of *dUbqn* induced ROS levels, which may lead to oxidative stress, and coffee honey attenuated this oxidative stress.

## Discussion

ALS is a fatal neurodegenerative disease. Due to progressive neurodegeneration, ALS leads to paralysis and death by respiratory failure 2–5 years after the onset of symptoms and there is currently no effective therapy. Mutations in the human *UBQLN2* gene are associated with ALS/FTD. Insects and insect-derived products have been used in traditional medicine for various diseases. In the present study, we utilized the ALS/FTD model fly targeting the *dUbqn* gene. In addition, since *Drosophila* larval NMJs contain mammalian homologues of glutamate receptors (GluRs) and also postsynaptic structures that resemble to mammalian models [64], we took this advantage to examine our insect products. It was found that among the tested 15 insect extracts and products, three treatments (coffee honey (1% v/v) from *A. cerana*, *A. dorsata* melittin (0.5 μg/ml), and wasp powder (2 mg/ml)) were the most effective at rescuing defects in the locomotive abilities of *dUbqn* knockdown flies. Furthermore, *dUbqn* knockdown flies fed with coffee honey (1% v/v) at least partially rescued synapse structural defects in the NMJs, improved learning ability, and reduced the accumulation of reactive oxygen species (ROS) caused by *dUbqn* depletion in the brain. In contrast, *A. dorsata* melittin and wasp powder exerted no suppressive effect on the defects in synapse structure and learning ability. Therefore, the improving effects of *A. dorsata* melittin and wasp powder on locomotive defects may be due to effects on some other pathways than the neuronal function such as energy production and muscular development. Further analyses are needed to clarify this point. From the results of our study we found that honey from coffee blossom effectively rescued the defects in behavior of mutant *dUbqn* larval flies. Coffee honey from *A. cerana* may be the potential candidate for the development of novel therapy for ALS/FTD patients.

Honey is a well-known natural product that is derived from honeybees. Worker honeybees collect nectar from blossoms and store them in a honey sack before they return to the hive. The nectar is then mixed with enzymes from honeybees to breakdown complex sugars in the nectar to simple sugars, such as glucose and fructose [65]. In addition to its sugar content, honey contains various molecules, including proteins and amino acids, trace amounts of enzymes, vitamins, and other substances, such as phenolic compounds [66]. Previous studies on honey clarified its biological properties, such as its antioxidant, anti-inflammatory, anti-bacterial, antiviral, and antiulcer activities as well as its anti-hyperlipidemic, anti-diabetic, and anti-cancer properties [14, 15, 67, 68]. Recent studies on Tualang honey, which is produced by giant honeybee (*Apis dorsata*) hives on Tualang trees located mainly in the north-western region of Peninsular Malaysia revealed that honey may exhibit significant activity against chronic cerebral hypoperfusion, which is partially responsible for Alzheimer’s disease [69]. Tualang honey has been reported to exert protective effects on learning and memory, accompanied by enhancements in the morphology of memory-related brain areas, increased levels of brain-derived neurotrophic factors, reduced brain oxidative stress, increased acetylcholine concentrations, and reduced acetylcholinesterase activity in brain homogenates [28, 70]. Thailand has various types of honey from various flowers, such as longan, coffee, lychee, sunflowers and wildflowers. According to previous report, coffee honey produced by *A. cerana* exhibited strong antioxidant activity (IC_50_ = 1.788 ± 0.329 mg/ml), reflecting stronger free radical-reducing activity [9]. In addition, coffee honey produced by *A. cerana* possessed the highest phenolic (1308.62 ± 27.83 mg GAE/kg) and flavonoid contents (0.152 ± 0.015 mg QE/g) [9]. Furthermore, the volatile compounds (e.g. 2-furanmethanol, butyryl lactone, phenyl methanol, anisaldehyde, anise alcohol, and 3,4,5-trimethyl-phenol) found in coffee honey produced by *A. cerana* may contribute to its antioxidant activity [16].

Since 2011, mutation in a human *UBQLN2* gene has been found to be associated with ALS/FTD (Amyotrophic Lateral Sclerosis/Frontotemporal dementia), the fatal neurodegenerative disease that progressively affected neuronal cells in both brain and spinal cord [49]. The lack of *UBQLNs* availability not only caused ALS/FTD, it also globally affected neuronal functions and makes the neurons more susceptible to various stressors. Previous studies have shown that knockdown of *dUbqn* flies led to detergent-insoluble ubiquitinated proteins and affected negative geotaxis in *dUbqn* knockdown flies [54, 71, 72]. Interestingly, *Drosophila* model of TDP-43 proteinopathy in motor neurons and glia cells showed the improvement of locomotive defects after high glucose consumption [73]. Indeed, TDP-43 proteinopathy was also expressed in *dUbqln* knockdown model [71]. Thus, it might be assumed that *dUbqn* knockdown flies could have some benefits of sugar consumption especially honey which contains high nutrition. However, our study revealed that, not all kinds of honey gave benefits to the *dUbqn* knockdown model, only coffee honey significantly has this ability. Oxidative stress is one of the mechanisms by which motor neuron death occurs [74]. The polyphenol ingredients of honey may quench the ROS that lead to neurotoxicity, aging, and the pathological deposition of misfolded proteins, including amyloid β [26].

Recently, we have found that pan-neuronal *dUbqn*-knockdown flies show significantly altered pre- and post-synaptic structural NMJ proteins by attenuating signals of Bruchpilot puncta and Glutamate receptor IIA clustering [72]. The *dUbqn*-knockdown flies also show a decrease in glutamate, an excitatory neurotransmitter, and increase in GABA, an inhibitory neurotransmitter, which is consistent with the phenotype of the *dUbqn*-knockdown flies [72]. Further analyses on the effect of coffee honey on distribution of these structural NMJ proteins together with the levels and distributions of neurochemicals would be interesting.

## Conclusion

In conclusion, we utilized the *dUbqn* knockdown model to test the effects of several insects and insect-related products and found that coffee honey effectively rescued the defects in locomotive ability, improved learning ability, and reduced the accumulation of reactive oxygen species (ROS) caused by *dUbqn* depletion in the brain. Furthermore, the coffee honey could also partially rescue the morphological defects in neuromuscular junction (NMJ) of the *dUbqn* knockdown flies. Several bioactive compounds or nutritive value in honey might be responsible for these effects. The present results suggest that the synergistic effects of coffee honey will ultimately contribute to the development of effective therapy for neurodegenerative disease. Thus, the coffee honey could be one of the promising insect-derived products which is worth to further investigate and develop in term of medicinal products. This discovery is significant because there is currently no cure or effective treatment options for ALS. Further study in other models is necessary to clarify and investigate deeply the underlying mechanisms.

## Supplementary information

**Additional file 1: Table S1 and S2.** The concentration of honeybee product and edible insect powder samples, and larval crawling path for *dUbqn* knockdown larvae.

**Additional file 2: Figure S1.** Quantification of *dUbqn* mRNA levels in control and untreated *dUbqn* knockdown flies. The *dUbqn* mRNA expression levels were normalized to *RpL32* mRNA levels (* *p* < 0.05).

## Data Availability

All data generated during this study are included in this article.

## Abbreviations

ALS: Amyotrophic lateral sclerosis
FTD: Frontotemporal dementia
NMJ: Neuromuscular junction
ROS: Reactive oxygen species
FALS: Familial cases of amyotrophic lateral sclerosis
FTLD: Frontotemporal lobar dementia
PBS: Phosphate-buffered saline
F: Forward primer
R: Reverse primer
SUC: Sucrose
OCT: 1-octanol
AM: n-Amyl acetate
LI: Learning index
FITC: Fluorescein isothiocyanate
HRP: Horseradish peroxidase
Dlg: Disc large
H_2_DCFDA: 2′,7′-dihydrofluorescein diacetate
IC_50_: Half maximal inhibitory concentration
QE: Quercetin equivalents
GAE: Gallic acid equivalent
VOCs: Volatile organic compounds
ADM: *Apis dorsata* melittin
ACM: *Apis cerana* melittin
AFM: *Apis florea* melittin
CH: Coffee honey
LH: Longan honey
TP: Tea pollen
FDRJ: Freeze-dried fresh royal jelly
Ci: Cicada powder
Si: Silkworm powder
Ba: Bamboo borer powder
Be: Honeybee larva powder
Gi: Giant water bug power
Cr: Crickets powder
Wa: Wasp power
Su: Subterranean ants powder
