# Supplementary material for: Honeybee products and edible insect powders improve locomotive and learning abilities of Ubiquilin-knockdown Drosophila
Source: BMC Complement Med Ther. 2020 Aug 31;20:267. doi: 10.1186/s12906-020-03054-8 (PMC7457359; doi:10.1186/s12906-020-03054-8)
Supplement: Supplementary file 1 — Additional file 1: Table S1 and S2. The concentration of honeybee product and edible insect powder samples, and larval crawling path for dUbqn knockdown larvae. [file 12906_2020_3054_MOESM1_ESM.pdf]

Title: Honeybee products and edible insect powders improve locomotive and learning abilities of *Ubiquilin*- knockdown *Drosophila*

Patcharin Phokasem[1,2], Salinee Jantrapirom[3,4], Jirarat Karinchai[5], Hideki Yoshida[3,6] Masamitsu Yamaguchi[3,6*] and Panuwan Chantawannakul[2,7*]

[1] Graduate School, Chiang Mai University, Chiang Mai 50200, Thailand
[2] Bee Protection laboratory, Department of Biology, Faculty of Science, Chiang Mai University. Chiang Mai 50200, Thailand
[3] Department of Applied Biology, Kyoto Institute of Technology, Matsugasaki, Sakyo-ku, Kyoto 606-8585, Japan
[4] Department of Pharmacology, Faculty of Medicine, Chiang Mai University, Chiang Mai, 50200, Thailand
[5] Department of Biochemistry, Faculty of Medicine, Chiang Mai University 50200, Thailand
[6] The Center for Advanced Insect Research, Kyoto Institute of Technology, Matsugasaki, Sakyo-ku, Kyoto 606-8585, Japan
[7] Environmental Science Research Center, Faculty of Science, Chiang Mai University, Chiang Mai, 50200, Thailand

*Correspondence: panuwan@gmail.com and myamaguc@kit.ac.jp

Supplementary table

Table S1: The concentration of honeybee product samples and larval crawling path for *dUbqn* knockdown larvae.

Sample	Abbreviation	Concentration	Path
Apis dorsata melittin	ADM	0.5 µg/ml	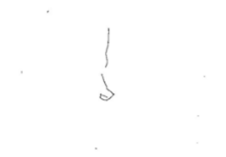
		2 µg/ml	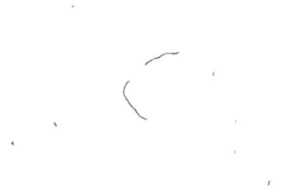
Apis cerana melittin	ACM	0.5 µg/ml	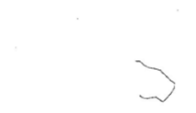
		2 µg/ml	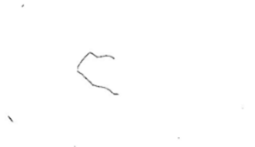

Table S1: The concentration of honeybee product samples and larval crawling path for *dUbqn* knockdown larvae (cont.).

Sample	Abbreviation	Concentration	Path
Apis florea melittin	AFM	0.5 µg/ml	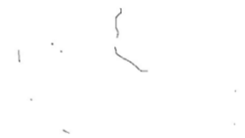
		2 µg/ml	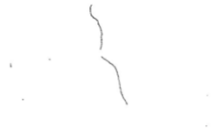
Coffee honey	CH	0.1% v/v	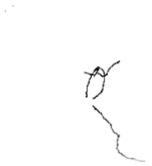
		1% v/v	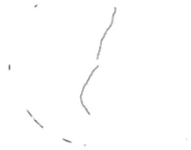
Longan honey	LH	0.1% v/v	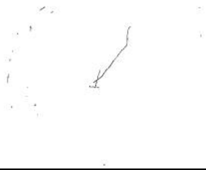
		1% v/v	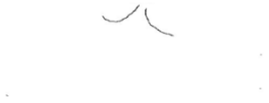
Tea pollen	TP	0.2 mg/ml	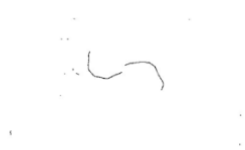
		2 mg/ml	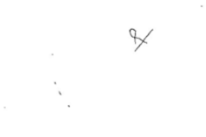

Table S1: The concentration of honeybee product samples and larval crawling path for *dUbqn* knockdown larvae (cont.).

Sample	Abbreviation	Concentration	Path
Freeze-dried royal jelly powder	FDRJ	0.2 mg/ml	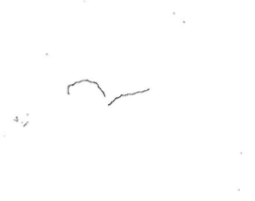
		2 mg/ml	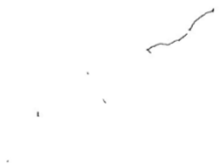

Table S2: The concentration of edible insect powder samples and larval crawling path for *dUbqn* knockdown larvae.

Scientific name	Common name	Abbreviation	Concentration	Path
Pompania sp.	Cicada powder	Ci	0.2 mg/ml	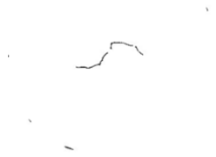
			2 mg/ml	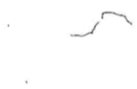
Bombyx mori	Silkworm powder	Si	0.2 mg/ml	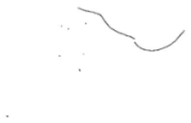
			2 mg/ml	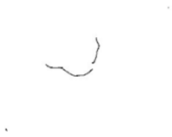
Omphisa fuscidentalis	Bamboo borer powder	Ba	0.2 mg/ml	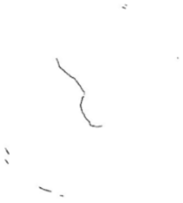
			2 mg/ml	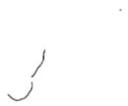

Table S2: The concentration of edible insect powder samples and larval crawling path for *dUbqn* knockdown larvae (cont.).

Scientific name	Common name	Abbreviation	Concentration	Path
Apis mellifera	Honeybee larva powder	Be	0.2 mg/ml	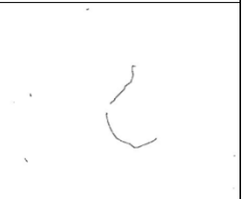
			2 mg/ml	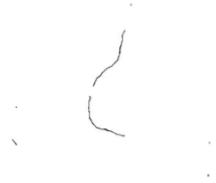
Lethocerus indicus	Giant water bug powder	Gi	0.2 mg/ml	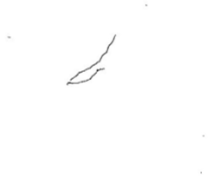
			2 mg/ml	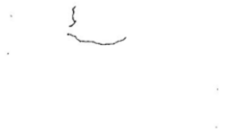
Gryllus bimaculatus	Cricket powder	Cr	0.2 mg/ml	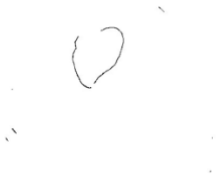
			2 mg/ml	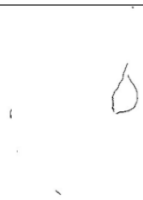

Table S2: The concentration of edible insect powder samples and larval crawling path for *dUbqn* knockdown larvae (cont.).

**Scientific name**	**Common name**	**Abbreviation**	**Concentration**	**Path**
Vespa affinis	Wasp powder	Wa	0.2 mg/ml	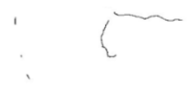
			2 mg/ml	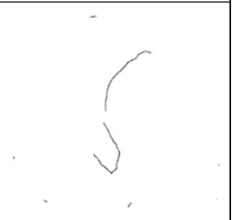
Carebara castanea	Subterranean ant powder	Su	0.2 mg/ml	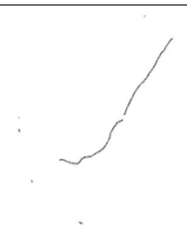
			2 mg/ml	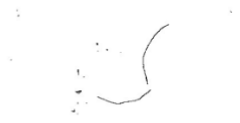
Untreated *dUbqn* knockdown larvae	-	-	-	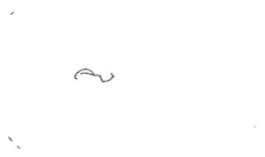
