# Supplementary material for: Honeybee products and edible insect powders improve locomotive and learning abilities of Ubiquilin-knockdown Drosophila
Source: BMC Complement Med Ther. 2020 Aug 31;20:267. doi: 10.1186/s12906-020-03054-8 (PMC7457359; doi:10.1186/s12906-020-03054-8)
Supplement: Supplementary file 2 — Additional file 2: Figure S1. Quantification of dUbqn mRNA levels in control and untreated dUbqn knockdown flies. The dUbqn mRNA expression levels were normalized to RpL32 mRNA levels (* p < 0.05). [file 12906_2020_3054_MOESM2_ESM.pdf]

Supplementary Figure S1

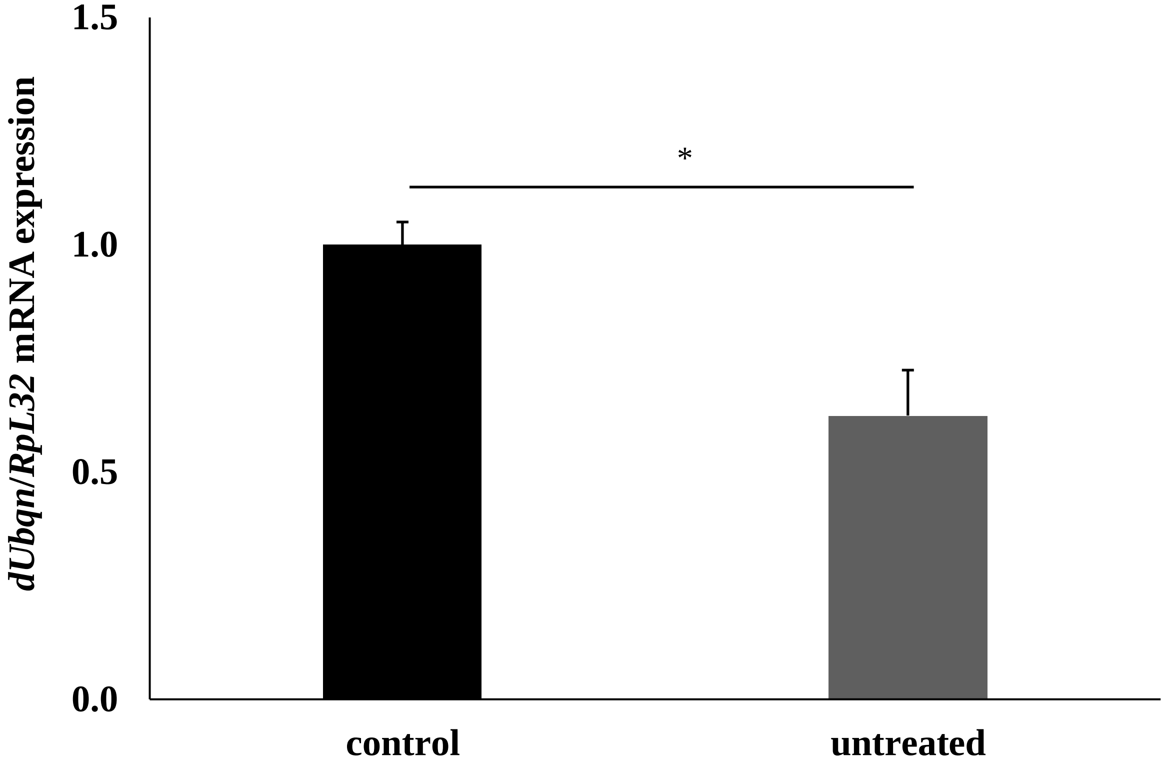

Supplementary Figure S1. Quantification of *dUbqn* mRNA levels in control and untreated *dUbqn* knockdown flies. The *dUbqn* mRNA expression levels were normalized to *RpL32* mRNA levels (* $p < 0.05$).
